# COVID-19 and prostate cancer: a complex scenario with multiple facets

**DOI:** 10.2144/fsoa-2021-0113

**Published:** 2021-11-23

**Authors:** Felice Crocetto, Luciana Buonerba, Luca Scafuri, Vincenzo Caputo, Biagio Barone, Antonella Sciarra, Antonio Verde, Armando Calogero, Carlo Buonerba, Giuseppe Di Lorenzo

**Affiliations:** ^1^Department of Neurosciences, Human Reproduction & Odontostomatology, University of Naples Federico II, Naples, 80131, Italy; ^2^Oncology Unit, Hospital ‘Andrea Tortora’, ASL Salerno, Pagani, Italy; ^3^Associazione O.R.A., Somma Vesuviana, Naples, Italy; ^4^Department of Experimental Medicine, University of Campania ‘Luigi Vanvitelli’, Naples, NA, Italy; ^5^Department of Advanced Biomedical Sciences, University of Naples Federico II, Naples, 80131, Italy; ^6^Department of Medicine & Health Science, University of Molise, Campobasso, Italy

**Keywords:** COVID-19, prostate cancer, SARS-CoV-2

COVID-19, the infectious disease caused by SARS-CoV-2, has claimed the lives of over 4.5 million people worldwide as of 18 September 2021 [1]. Besides having a multitude of social, economic and sanitary consequences [2–4], which have aggravated pre-existing threats to public health, such as those related to pollution [5], the global pandemic has profoundly affected several aspects of cancer care, including doctor–patient relationships, [6], access to therapy and physicians’ therapeutic choices [7]. This has ultimately negatively influenced cancer-specific mortality [8]. Conversely, COVID-19 is associated with higher mortality rates in cancer versus non-cancer patients [9].

In the particular case of prostate cancer, the second most frequently diagnosed cancer in men, the COVID-19 pandemic has had a negative impact both on early diagnosis by reducing participation to screening programs [10] and on time from diagnosis to surgery/radiotherapy [11], which may translate in a worse prostate-cancer-specific mortality in the next years [12]. In spite of the hurdles associated with diagnosis of prostate cancer [13], population-based prostate-specific antigen (PSA) screening has proven to reduce prostate-cancer-specific mortality, although over 1000 men have to be screened to prevent a single death from prostate cancer [14]. A retrospective study conducted at the University Hospitals of Verona was designed to identify all test requests for total PSA and vitamin D for outpatients from 10 December 2016 to 10 December 2020 and compare the weekly requests for these tests between 25 February and 9 December 2020, to those sent during the same period of the past 4 years (2016–2019). Of note, the weekly rate of test requests was consistent across the years, but a sharp decline was recorded during the lockdown period (between 10 March and 17 May 2020), with median decrease of 76% for vitamin D and 62% for total PSA, respectively [10]. Another retrospective observational study conducted in 2020 at a high-volume center was designed to assess the impact of COVID-19 on time to surgery of patients on the urology surgical waiting list. In the overall cohort of 350 patients (including 20 patients with prostate cancer), the mean time of 97.33 days on the waiting list was reported, which was significantly longer compared with that reported in 2019 [11]. In a population-based study exploring patterns of radiotherapy use, mean weekly radiotherapy courses decreased by 19.9% in April, 6.2% in May and 11.6% in June 2020 compared with corresponding months in 2019, with the largest reduction reported for prostate cancer (77.0% in April) [15]. The delayed diagnosis caused by the COVID-19 pandemic has been estimated to result in a 17.2% increase in prostate cancer deaths in the years 2022–24 using a novel modeling approach [12]. While a meta-analysis of six retrospective studies including a total of 50,220 patients suggested that androgen deprivation therapy may not be associated with an increased risk of being infected with SARS-CoV-2 [16], the number of previous systemic lines of treatment may influence prognosis. In a retrospective multicenter study including 34 patients with metastatic castration-resistant prostate cancer who were observed at the time of COVID-19 diagnosis, 17 patients (50.0%) had recovered, 13 patients (38.2%) had died and four (11.7%) were still positive for SARS-CoV-2, after a median follow-up time of 21 days. Importantly, the multivariate analysis of this retrospective cohort showed that the number of previously administered antineoplastic treatments for metastatic castration-resistant prostate cancer was significantly associated with COVID-19 mortality. Although one COVID-19 case was reported in a patient receiving the hormonal treatment enzalutamide [17] with no apparent enzalutamide-related detrimental effects on COVID-19 prognosis, there are no data suggesting that patients on novel oral androgen-receptor targeting agents (such as enzalutamide or abiraterone [18]) may be safer compared with chemotherapy agents such as cabazitaxel [19] in the case of SARS-CoV-2 infection. The lack of clinically significant bone marrow toxicity and their oral route of administration have made androgen-receptor axis targeting agents a more attractive therapeutic option compared with intravenous taxanes during the COVID-19 pandemic. Finally, it must be considered how COVID-19 can affect accuracy of imaging techniques. In a retrospective multicenter study including nine men with COVID-19 who underwent ^68^Ga-PSMA-11-PET/CT for prostate cancer [20], all of them showed different grades of abnormal ^68^Ga-PSMA-11 uptake in the lungs, but only a single patient actually had lung metastasis. This finding highlights the potential confounding effect of COVID-19 on prostate cancer staging using nuclear medicine techniques.

Overall, the findings discussed here show how COVID-19 has deeply affected all aspects of prostate cancer care, including early diagnosis, treatment and staging, with negative consequences that are not fully predictable and understandable at the present time. On the other hand, it is foreseen that SARS-CoV-2 will continue to circulate despite mass vaccination programs, so a continued effort to analyze its effects on prostate cancer care is mandatory. Physicians treating prostate cancer should consider preferring oral compared with intravenous agents and performing remote visits, if feasible. Also, they should be aware that COVID-19 may affect accuracy of some imaging techniques such as PSMA (prostate specific membrane antigen) PET. On the other hand, research should focus on the potential interaction of COVID-19 with available treatments against prostate cancer, which remains largely ignored. As an example, patients with localized prostate cancer recovering from COVID-19 may represent a clinically significant population requiring a specific treatment algorithm regarding the use of surgery versus radiotherapy. Also, patients with metastatic prostate cancer with a history of symptomatic COVID-19 might suffer from more severe adverse events associated with chemotherapy. Observational prospective and retrospective studies designed to assess the impact of COVID-19 on treatment efficacy and toxicity remain a compelling need regardless of the success of vaccination campaigns in SARS-CoV-2 eradication in the complex scenario caused by this vicious and unprecedented pandemic.
